# Acid base homeostasis and serum bicarbonate concentration in syndrome of inappropriate anti-diuretic hormone secretion (SIADH) with hyponatremia

**DOI:** 10.3389/fendo.2023.1321338

**Published:** 2023-12-05

**Authors:** Manoocher Soleimani

**Affiliations:** ^1^ Department of Medicine, University of New Mexico School of Medicine, Albuquerque, NM, United States; ^2^ Research Services, New Mexico Veterans Health Care System, Albuquerque, NM, United States

**Keywords:** SIADH, hyponatremia, acid base balance, bicarbonate, collecting duct, AVPR1a, intercalated cells, H^+^-ATPase

## Abstract

The Syndrome of Inappropriate ADH secretion (SIADH) presents with excess ADH release caused by a range of conditions; including pneumonia, brain tumors, certain lung cancers, and diseases of the hypothalamus. It presents with significant reduction in both sodium and chloride concentrations in the blood. However, reports examining the acid base status indicate a normal serum bicarbonate concentration and systemic acid base homeostasis. The mechanisms for the absence of abnormalities in acid base homeostasis remain speculative. This mini review is highlighting the recent advances in renal molecular physiology to provide answers for the maintenance of acid base status and serum bicarbonate in a physiological range.

## 1 Introduction

The Syndrome of Inappropriate Anti-Diuretic Hormone secretion (SIADH) is a frequent clinical encounter with significant morbidity and mortality in hospital admissions. At its core, this disorder presents with excess ADH due to the inability of the body to suppress its secretion. It is caused by a range of conditions, including pneumonia, brain tumors, certain lung cancers, and diseases of the hypothalamus (1–3).

There are hardly any studies examining the mechanism of acid base homeostasis in SIADH despite the significant reduction in both sodium and chloride concentrations in the blood. All reports refer to normal serum bicarbonate levels and by inference a balanced acid base status in SIADH. In this short commentary, we have discussed the physiological and molecular pathways that may be playing a significant role in maintaining a normal acid base homeostasis in SIADH.

## 2 Case presentation

A 62-year-old man was brought to an emergency room with altered mental status. Patient has a history of a stable lung mass, but has refused any workup.

Blood chemistries on admission showed the following: Na^+^112 meq/l, Cl^-^ 79 meq and BUN 5 mg/dl. The urine Na^+^ was 65, urine K^+^ was 42 and the urine osmolality was 420 mosm/l. Serum bicarbonate concentration was 26 mEq/l.

## 3 Discussion

The above presentation is typical of SIADH-induced hyponatremia. Excess ADH causes the water absorbing channel, AQP-2, to be translocated to the apical membrane of kidney principal cells; therefore, enhancing water absorption and impairing water excretion (4). This results in the dilution of serum Na^+^ and Cl^-^ and the concentration of urine osmolality, respectively. Patients can present with profound hyponatremia, hypochloremia, and frequently with low uric acid.

**Figure 1:**
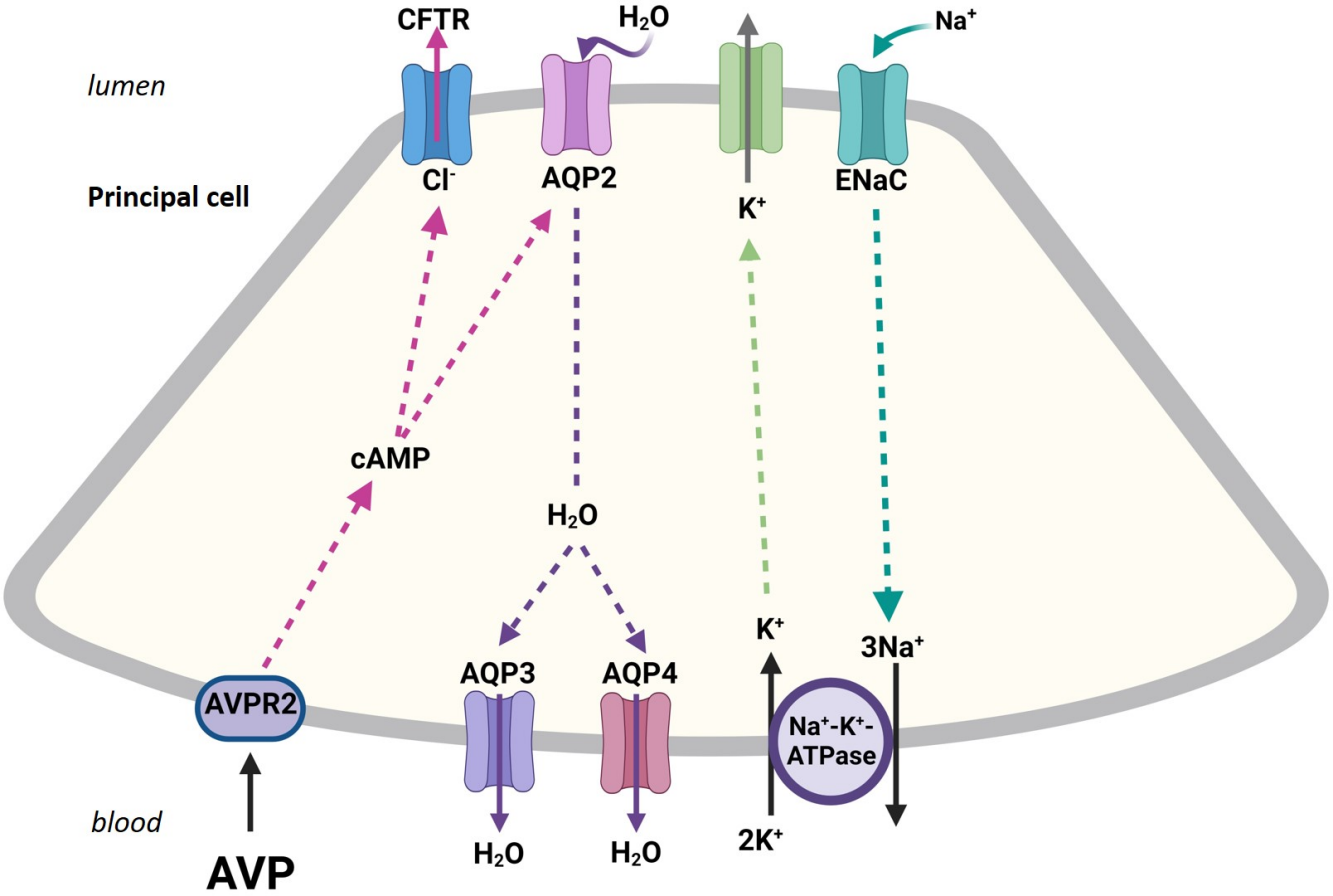
The schematic diagram exhibits the binding of ADH (AVP) with V2R resulting in enhanced intracellular cAMP which leads to the insertion of AQP-2 water channel into the apical membrane of principal cells; therefore, increasing water absorption. The absorbed water exits the cell via the basolateral AQP-3 and AQP-4 water channels.

Schematic diagram in **Figure 1** depicts the binding of ADH with the vasopressin receptor isoform 2 (V2R) which results in enhanced intracellular cAMP and AQP-2 insertion into the apical membrane of principal cells; therefore, increasing water absorption.

Despite a significant reduction in serum Na^+^ and Cl^-^ concentration, all clinical studies demonstrate a normal serum bicarbonate level consistent with the absence of any acid/base disturbance in individuals with SIADH (5). The exception to this statement is found in SIADH associated with Addison Disease which can present with hyponatremia and metabolic acidosis (6).

While there are no concrete studies to address the mechanism of static acid base homeostasis in SIADH-induced hyponatremia, possibilities such as an initial dilutional (volume expansion) acidosis stimulating a restorative acid excretion is plausible. However, there are no studies available that point to an initial metabolic acidosis in SIADH-induced hyponatremia.

A reappraisal of the published studies, as well as recent investigations on the role of ADH on acid base balance, may provide some strong clues on the mechanism of stable serum HCO_3_^-^ concentration in SIADH.

### 3.1 ADH can stimulate H^+^ secretion via apical H^+^-ATPase in kidney A-intercalated cells

**Figure 2:**
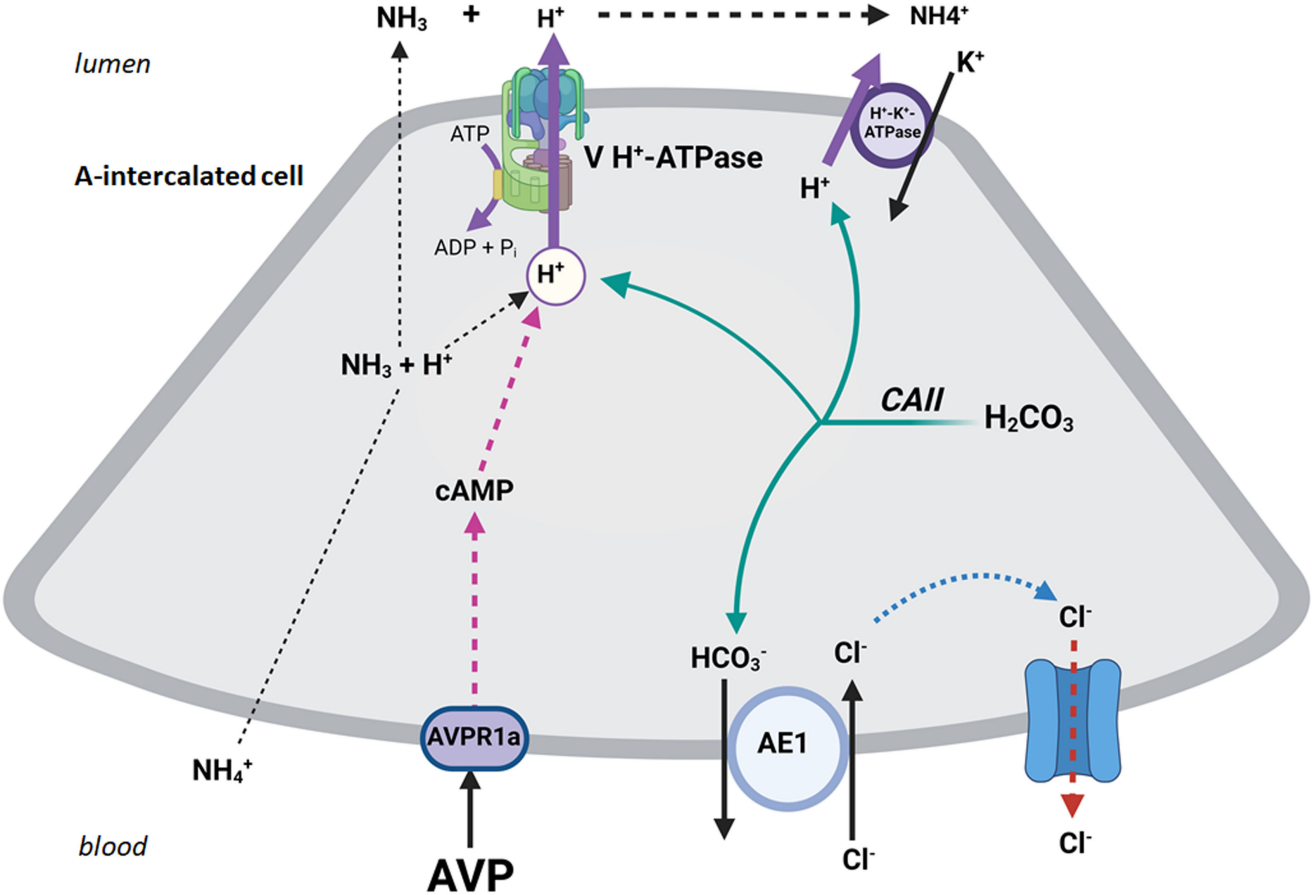
The schematic diagram exhibits the binding of ADH (AVP) with the basolateral V1R (AVPR1a) in A-IC cells resulting in the translocation of H^+^-ATPase to the apical membrane and stimulation of H^+^ secretion into the lumen of the collecting duct.

Aside from the V2 receptor (AVPR2) which is expressed on the basolateral membrane of principal cells (**Figure 1**) and is inhibited by tolvaptan (5), recent studies demonstrated the presence of V1Ra (AVPR1a) receptor on the basolateral membrane of A-intercalated cells. See **Figure 2** for detail. These studies further indicate that ADH addition to the interstitial compartment of perfused collecting duct enhances the translocation of H^+^-ATPase to the apical membrane and stimulation of H^+^ secretion into the lumen of the collecting duct (7, 8).

### 3.2 Hypotonicity can directly stimulate H^+^-ATPase-mediated H^+^ secretion in cultured kidney collecting duct cells

Independent of the excess ADH-mediated effect, hypotonicity can directly stimulate H^+^-ATPase in cultured kidney IMCD cells (9). These studies demonstrate a direct stimulatory effect of hypotonicity on H^+^ secretion via H^+^-ATPase in cultured kidney collecting duct cells (9). The effect is rapid in onset (less than few minutes) and is likely mediated through the activation of existing H^+^-ATPase molecules in the plasma membrane of cultured kidney cells.

### 3.3 Hypotonicity and ammoniagenesis

Activation of H^+^-ATPase by itself may not significantly enhance bicarbonate absorption in the collecting duct or increase blood bicarbonate concentration (specifically if the urine pH is already less than 6). However, H^+^-ATPase activation is necessary for retaining the new bicarbonate which is generated consequent to enhanced ammoniagenesis.

### 3.4 Is there any evidence of enhanced ammoniagenesis in hyponatremia?

Studies by Halperin and Ching show that hyponatremia can enhance ammoniagenesis in mammalian kidneys (10). It is worth mentioning that there are no studies examining urinary NH_4_^+^ excretion in patients with SIADH and hyponatremia.

In addition to the collecting duct, ADH and osmolality are two critical factors capable of regulating bicarbonate absorption in the medullary thick ascending limb (MTAL). Detailed studies in perfused tubules demonstrated that hypertonicity markedly inhibits (11, 12); whereas, hypotonicity stimulates bicarbonate absorption in the MTAL, the latter is due to activation of the apical Na^+^/H^+^ exchanger isoform NHE3 (13). The presence of vasopressin inhibited the hypotonicity-stimulated bicarbonate absorption via NHE3 in MTAL (13). These results strongly indicate that enhanced luminal H^+^ secretion via NHE3 and the consequent bicarbonate absorption in MTAL by hypotonicity do not contribute to maintaining acid base homeostasis in patients with SIADH since both are inhibited by AVP.

Lastly, it is worth mentioning that the circulating aldosterone level is not suppressed in individuals with SIADH and hyponatremia. Studies examining the effect of aldosterone on acid secretion in the kidney OMCD indicate that the stimulatory effect of aldosterone on H^+^ secretion (presumably via H^+^-ATPase and/or H^+^-K^+^ ATPase) requires a functional V1a receptor in the intercalated cells (14).

## 4 Conclusion

Hypotonicity and ADH can stimulate H^+^-ATPase and in coordination with enhanced ammoniagenesis maintain the systemic acid base status and serum bicarbonate concentration at a normal level in hypotonic SIADH states. Examining urine NH_4_^+^ excretion in individuals with SIADH and hyponatremia, as well as determining the expression of ammoniagenesis enzymes and H^+^-ATPase in rodents with ADH injection and excess water consumption (mimicking SIADH-induced hyponatremia), can shed light on this issue.

## Author contributions

MS: Conceptualization, Formal Analysis, Funding acquisition, Investigation, Resources, Visualization, Writing – original draft, Writing – review & editing.
